# Driver Drowsiness Detection: A Machine Learning Approach on Skin Conductance

**DOI:** 10.3390/s23084004

**Published:** 2023-04-15

**Authors:** Andrea Amidei, Susanna Spinsante, Grazia Iadarola, Simone Benatti, Federico Tramarin, Paolo Pavan, Luigi Rovati

**Affiliations:** 1 Dipartimento di Ingegneria “Enzo Ferrari”, Università di Modena e Reggio Emilia, Via Pietro Vivarelli 10, 41125 Modena, Italy; andrea.amidei@unimore.it (A.A.);; 2 Department of Information Engineering, Polytechnic University of Marche, 60131 Ancona, Italy

**Keywords:** driver monitoring, drowsiness detection, skin conductance, galvanic skin response, machine learning, wearable devices, active assisted living

## Abstract

The majority of car accidents worldwide are caused by drowsy drivers. Therefore, it is important to be able to detect when a driver is starting to feel drowsy in order to warn them before a serious accident occurs. Sometimes, drivers are not aware of their own drowsiness, but changes in their body signals can indicate that they are getting tired. Previous studies have used large and intrusive sensor systems that can be worn by the driver or placed in the vehicle to collect information about the driver’s physical status from a variety of signals that are either physiological or vehicle-related. This study focuses on the use of a single wrist device that is comfortable for the driver to wear and appropriate signal processing to detect drowsiness by analyzing only the physiological skin conductance (SC) signal. To determine whether the driver is drowsy, the study tests three ensemble algorithms and finds that the Boosting algorithm is the most effective in detecting drowsiness with an accuracy of 89.4%. The results of this study show that it is possible to identify when a driver is drowsy using only signals from the skin on the wrist, and this encourages further research to develop a real-time warning system for early detection of drowsiness.

## 1. Introduction

Drowsy driving is one of the known causes of road accidents, which account for approximately 1.35 million deaths each year, according to the 2018 World Health Organization (WHO) report [1]. A recent review of motor vehicle crashes in the United States in 2020, published by the National Highway Traffic Safety Administration (NHTSA), states that while the number of police-reported crashes decreased by 22% from 2019 to 2020, the number of people killed in traffic accidents increased by 6.8% over the same period [2]. In Europe, according to 2021 road safety statistics [3], 44 road deaths per million inhabitants were reported, which is an increase of 5% compared to 2020 but a decrease of 13% compared to 2019 before the pandemic.

Several studies show that deaths and injuries from traffic crashes are primarily caused by human factors, including distraction and drowsiness, strictly related to driver fatigue. Specifically, on the basis of extensive collections of statistical data on traffic accidents, the NHTSA found that many crashes associated with drowsy driving involve a single vehicle, with no passengers other than the driver, running off the road at a relevant speed with no evidence of braking. In most cases, drowsy driving crashes occur on rural roads and highways. Similarly, the road safety thematic report on fatigue published by the European Commission’s European Road Safety Observatory in 2021 [4] estimates that fatigue is a contributing factor in 15% to 20% of serious crashes, although individual studies report quite different results. A meta-analysis of 14 different studies [5] found that fatigued drivers have a 29% increased risk of crashing.

In view of the situation described in the above-mentioned reports, research in the field of driving safety is moving toward the development of automatic techniques for detecting the driver’s drowsy state, through the appropriate selection of sensor technologies and data processing approaches based on Machine Learning (ML) and Artificial Intelligence (AI) in order to alert the driver as soon as possible [6]. Automatic fatigue detection systems aim to warn drivers when they are getting tired. Since 1 September 2020, in Europe, the presence of a driver drowsiness and attention warning system has become mandatory for all vehicle categories and included in the motor vehicle type approval requirements. It is possible to find solutions that mainly use sensors that monitor the vehicle’s operation or the driver’s condition. In the latter case, cabin-mounted or wearable sensors can be used.

Existing safety technologies can detect the effects of drowsy driving and warn the driver by monitoring the vehicle’s lane departure attitude combined with analysis of steering wheel rotation [7]. These warning systems are already embedded in vehicles manufactured by Ford Motor Company and Volkswagen [8,9]. However, these systems detect the sleep status of the driver, not the drowsiness. Other solutions use cameras installed in the vehicle cabin to track the driver’s eye movements, facial expressions, and head position [10], such as the driver attention monitoring system developed by Lexus and Toyota [11]. Camera-based solutions [12] are more reliable in providing timely detection of drowsy behavior, but on the one hand, they require one or more cameras installed in the vehicle cabin (which is not yet common), and on the other hand, they may suffer from several limitations or constraints due to the operating conditions (e.g., different and variable lighting scenarios or possible occlusions of the driver’s face, such as glasses or sunglasses). For these reasons, researchers and car manufacturers have explored different and innovative solutions. In addition to approaches using image-based and vehicle-based data, biosignals can be collected from the driver and processed to detect drowsiness and possibly merged with other sensor data to implement hybrid drowsiness detection systems. In this scenario, an innovative approach is the use of skin conductance (SC) or galvanic skin response. SC signals can be analyzed, in fact, through non-invasive integrated sensor platforms.

Based on these premises, the aim of this manuscript is to investigate the ability of the SC signal collected from the wrist to detect the drowsiness of a driver through the use of a wearable device, which in this study has been selected as the Empatica E4. This paper technically extends the investigations of a preliminary study by the same authors [13]. In this enhanced and extended version, the classification accuracy of the SC signals collected by the wearable device is further assessed, providing a comparison with the classification accuracy of the SC signals collected by a reference instrument, namely the ProComp Infiniti DAQ [14]. For a proper comparison, signals are obtained simultaneously by the wearable and the ProComp from the same subjects. The results show that the signals from the wearable device need to be appropriately resampled, filtered, and cleaned of motion artifacts in order to achieve a classification accuracy comparable to that obtained from the signals acquired by the reference instrument. Finally, the tested ML algorithms show a classification accuracy above 89%. This is an important result to support experiments to detect driver drowsiness using a simple wrist-worn device, independent of other technological equipment on board the vehicle.

The paper is structured as follows. Section 2 provides an analysis of the state-of-the-art about drowsiness detection systems based on physiological signals. Background concepts about the acquisition of the SC and the removal of motion artifacts are given in Section 3. In Section 4, the study methodology is described, including the description of the driving simulator, the acquisition devices, data analysis and the test procedure. Preliminary results and filtering improvements are given in Section 5. In Section 6, the comparison between the two devices and the final results are discussed. Finally, conclusions and perspectives for future studies are explained in Section 7.

## 2. Drowsiness Detection Based on Physiological Signals

A recent review by Albadawi et al. [15] presents several systems that use different measures to track and detect drowsiness. The main challenges for the use of biosignal-based systems are related to the characteristics of the devices used and the hardware setup. As a condition affecting driver behavior, drowsiness is related to the activity of the Autonomous Nervous System (ANS), which is reflected in physiological changes [16]. Nowadays, such changes can be monitored by means of comfortable wearable devices available either on the market or as research prototypes under different designs, such as wristbands [17,18] and rings [19]. In drowsiness studies based on biomarker collection reflecting ANS activity, the electroencephalogram (EEG) is commonly used as a reference signal, often in conjunction with the electrocardiogram (ECG). Furthermore, features of photoplethysmogram signals [20] or signals related to ECG—heart rate (HR), heart rate variability (HRV) and phonocardiogram [21,22,23]—can be considered.

In [24], electromyogram (EMG), hand pressure, and SC signals are measured in a minimally invasive manner using an integrated sensing platform mounted on a steering wheel sleeve. This allows the proposed sensing platform to be installed in any existing vehicle, enabling convenient and widespread driver monitoring. In fact, as already mentioned in the Introduction, if we focus on the skin, the ANS activity can be studied by analyzing SC, which is a biomarker that varies as a consequence of sweat gland secretion. The SC signal can be decomposed into two main components: a tonic component that varies slowly over time, also known as Skin Conductance Level (SCL) [25], and a phasic component characterized by rapid changes in signal amplitude, also known as Skin Conductance Response (SCR) [26], which is typically associated with stimuli-related events. The process of exosomatic SC acquisition is minimally invasive and can be performed during normal daily activities, especially when using wearable devices: data collection does not require controlled laboratory settings, and the portability of the devices makes them affordable. However, evidence from the literature shows that SC signals collected by wearable devices inevitably show limitations in terms of their quality compared to those acquired by laboratory equipment and DAQ (Data Acquisition) boards [27]. For example, movements performed by subjects during daily activities can strongly affect the signal with noisy artifacts. Therefore, appropriate processing steps must be applied to SC signals collected from a wrist-worn device to make them suitable for the reliable classification of drowsiness by ML algorithms.

The collection of multiple signals and biomarkers to achieve reliable drowsiness detection has been exploited in the literature. For example, Awais et al. [21] proposed the combined acquisition and processing of electroencephalographic (EEG) and electrocardiographic (ECG) signals to discriminate between alert and drowsy states, achieving an accuracy of 80.90% with the Support Vector Machine (SVM) ML classifier. Similarly, in [23], the authors present a drowsiness detection model that fuses physiological (from ECG, respiration sensor, and camera), postural, and vehicle information. Recently, Arjunan et al. [28] proposed a monitoring system that uses the combination of data from multiple wearable sensors, namely blood pressure, heart rate, blood oxygen, body temperature, EEG, and electromyography (EMG) sensors, to detect the status of the driver.

The SC signal, either alone or combined with other physiological signals, has been used to detect a subject’s drowsiness, with or without using ML algorithms to perform automatic classification of the drowsy state. For example, a wearable device capable of detecting the subject’s SC has been designed by the authors and presented in [29]. The signal collected by the proposed device shows obvious and distinguishable physiological variations that occur with the drowsy driving of the subject performing the test. Similarly, in [30], significant changes of the SC are visible when the subject falls asleep: this effect can be exploited as a meaningful property to identify an increasing drowsiness. In both studies, no classification of driver status was performed. A recent review of ML techniques applied to arousal classification from SC signals [31] shows that SVMs and Artificial Neural Networks (ANNs) emerge among the supervised learning methods, as they are able to provide high accuracy values. On the contrary, unsupervised methods are not found in the literature as a viable way to classify arousal from SC signals. This finding may be helpful for the correct selection of the classification algorithm to be applied in the detection of driver drowsiness. Wang et al. [32] combined SC with pulse oximetry and respiration signals to predict drowsy driving, using a Random Forest (RF) classifier combined with Hilbert–Huang transforms to maximize prediction accuracy. In [33], SC and Blood Volume Pulse (BVP) signals are jointly acquired and processed to detect and classify driver drowsiness by testing different ML algorithms.

In a recently published study [34], behavioral and physiological measures collected by a multi-sensor system using a camera to capture facial features from videos and a Galvanic Skin Response (GSR) sensor applied to the subject’s skin are combined to detect drowsiness. The proposed hybrid system can achieve an accuracy of 91% in identifying the transition of a driver’s status from awake to drowsy in all conditions tested with a driving simulator. Similarly, Horng et al. [35] and Choi et al. [36] focused on the physiological prediction of drowsiness by using a wrist-worn multi-sensor device that collects SC, EEG, and ECG data to monitor the driver’s stress, drowsiness, and fatigue states. In both works, ML classifiers were used to recognize the driver’s status. High classification accuracy was achieved by implementing the Support Vector Machine algorithm combined with an ensemble method for the multi-class classification problem. High classification performance was also achieved by [35], who focused on physiological prediction of drowsiness by measuring GSR, eye movements, HR, and brain waves from multiple wearable sensors. However, due to the bulky setup (e.g., electrodes for EEG placed on the head, those for ECG on the chest, and those dedicated to SC on the fingers/wrist), multimodal systems prove to be uncomfortable and intrusive arrangements for real-life adoption in driving situations. In addition, the use of multiple signals (from both wearable and ambient sensors) increases the complexity of the acquisition system, which could affect the data analysis procedure; consequently, the assessment of the driver status becomes more time-consuming, possibly leading to a delay in warning generation and reduced safety for the driver.

Detecting driver fatigue is a challenging task: any fatigue detection method becomes feasible if it is able to process data as quickly as possible while providing accurate results. Hybrid methods, based on multi-sensor systems and on the fusion of features extracted from signals of different types, can ensure better classification accuracy but also increase the complexity of the system. Therefore, it is of interest to investigate whether a single signal, such as the SC collected by a wrist-worn device, can be properly processed to achieve acceptable sleepiness classification accuracy.

## 3. Background Concepts

This section provides background concepts on the skin conductance signal and issues affecting its measurement for a better understanding of what is presented later on. A brief overview of common artifact removal methods is also provided.

### 3.1. Skin Conductance

According to [37], SC may be defined as the phenomenon that the skin temporarily becomes a better conductor of electricity due to a change in sweat secretion, when either external or internal stimuli occur that are physiologically arousing. As such, SC is an electrical signal measured indirectly according to Ohm’s law, and it is represented by the variation in the electrical conductivity of the skin.

It is important to emphasize that the physiological response that generates the SC variation is a time-limited phenomenon; therefore, time plays an important role in the experimental phase, both in the administration of the stimulus and in the measurement of the induced SC variation. Sweat secretion cannot be consciously controlled because it is driven by the ANS. The ANS includes sympathetic (SNS) and parasympathetic (PSNS) branches: the former represents a rapid response system for immediate motor action; therefore, it is also related to the fight or flight response. Increased sympathetic activity is associated with physical indicators of autonomic arousal, such as increased heart rate, respiratory rate, blood pressure, and sweating. The unconscious processes of sweat secretion reflect changes in arousal and are therefore controlled solely by the SNS. Arousal, in turn, has been found to be a strong predictor of attention and memory both in mental workload experiments and in simulated driving [38,39].

SC can be measured by endosomatic or exosomatic methods. In the former, an electrical potential difference is measured across the palmar and plantar skin in the absence of an applied external voltage or current. Typically, a single electrode is placed at the active site and a reference electrode is placed at a relatively inactive site, such as the forearm. The latter methods can be completed in DC with a constant small voltage (e.g., 0.5 V) applied between two electrodes placed on the skin surface. Using Ohm’s law, the skin resistance (SR) or its reciprocal SC can be derived. In DC measurements, electrode polarization can occur and affect the accuracy of SC measurement. In the AC modality, which avoids electrode polarization, skin impedance or its reciprocal (skin admittance) is measured.

Regardless of the specific method used to measure SC, several quantities and conditions can affect the quality of the measured data, among which it is worth mentioning: ambient temperature and humidity (possibly influencing the subject’s sweating); correct use and positioning of the electrodes; and body movement. The latter is an issue that has been addressed in the literature by different approaches, which are summarized in the following section.

### 3.2. Motion Artifacts and Correction Approaches

The increasing use of wearable devices for a long-term and minimally invasive acquisition of SC in real-life scenarios (e.g., out of ambulatory and controlled settings) makes the signal more vulnerable to noise and artifacts. The latter are typically due to electronic noise or changes in the skin surface-recording electrode contact because of varying pressure related to the measurement site chosen (such as the ventral wrist), excessive movement (depending on the specific situation under which the signal is acquired), or device adjustment. SC artifacts can be easily misunderstood as SCRs, i.e., stimuli-related variations of the SC, so it is evident that effective approaches to remove or mitigate artifacts in the SC signal are needed for a correct extraction of meaningful information from the signal itself [40]. At the same time, because SC does not exhibit periodicity (differently from other physiological signals) and also features a quite remarkable intra- and inter-subject variability, the issue of correctly identifying motion artifacts remains still open.

Heuristic removal methods are based on visual inspection of the SC signal, looking for atypical portions of it, compared to models derived from Boucsein’s analyses [37,41,42]. Such methods, however, do not generalize effectively beyond the contexts in which they have been designed based on signals acquired under specific conditions. Similar limitations hold for more recent methods based on curve fitting or sparse recovery [43], as they also rely on a model used to generate an artifacts-free version of the signal against which the one collected in the field is compared.

Common non-heuristic approaches for motion artifacts removal in SC signals exploit low-pass filtering or exponential smoothing [44]. These methods are effective in removing high variations in the SC signal but do not perform well when high-amplitude changes occur in a very short time in the signal because of the subject’s motion. On the other hand, methods exploiting machine learning and features-based classification have proven to be capable of handling spiky motion artifacts featuring huge changes in signal amplitude, but then they perform poorly on different types of noise contributions possibly present within the signal. For example, Taylor et al. proposed the use of a Support Vector Machine (SVM) as the selected classifier for the automatic detection of motion artifacts in EDA signals [40], while simple decision rules were applied in [45] but with limited generalization. In [46], three unsupervised learning methods and a threshold selection process were jointly applied to remove artifacts in EDA signals.

Motion artifacts removal methods based on Wavelet Transforms have gained popularity in different fields dealing with noisy physiological signals, and they have been in the literature for several years thanks to their time-frequency localization capability. Regarding the removal of motion artifacts from SC or EDA signals, Chen et al. [40] proposed the use of a Stationary Wavelet Transform (SWT), modeling the Wavelet coefficients as a Gaussian mixture distribution, to match both the SCL and SCRs. Their method could remove motion artifacts better than previously proposed solutions while keeping safe the noise-free portions of the signals. This method, combined with a specific modeling of the Wavelet coefficients, was used in a previous study from the authors to obtain artifacts-free SC signals [13]. In this work, the same filtering approach is modified and optimized by analyzing the impact that artifacts removal has on the automatic classification algorithms aimed at identifying the driver’s drowsiness.

## 4. System and Methods

The objective of the proposed analysis is to investigate the feasibility of an automatic detection of driver drowsiness by analyzing the SC signal alone. To this end, the driver’s SC signal is acquired with the Empatica E4 wearable device, as it would emulate a realistic scenario where drivers can wear their smartwatch to monitor physiological parameters. This device will therefore be the target device of this study.

The subsequent data analysis is performed first in a MATLAB environment and then using the WEKA tool [47] to evaluate the drowsiness classification accuracy achievable using different ML classifiers. In the following, all the required components of the acquisition system are presented.

### 4.1. Driving Simulator and Acquisition Devices

**Figure 1:**
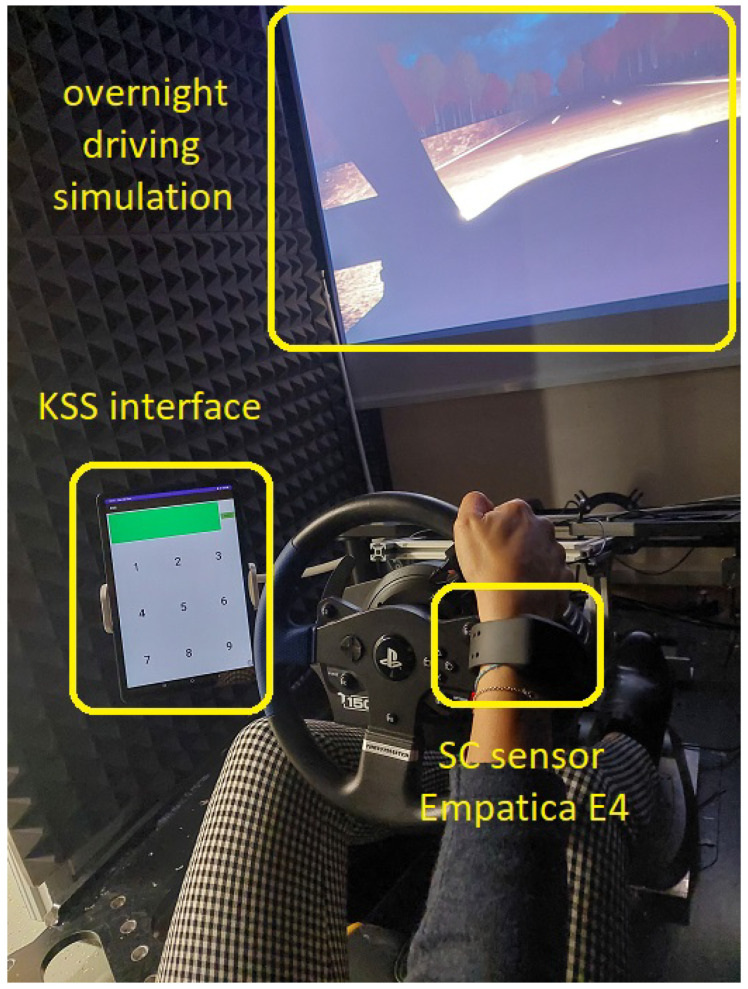
Experimental setup including: driving simulator, monitor presenting the overnight scenario, Empatica E4 device on the wrist, and tablet with KSS graphical interface.

With the aim to collect SC signals from subjects realistically engaged in a driving task, experiments are conducted in a driving simulator hosted at the Department of Engineering of the University of Modena and Reggio Emilia, as shown in Figure 1. An overnight driving path is selected as the simulated scenario, which is realized as a three-lane highway with no traffic and a length of 80 km. In order to reduce the influence of random effects/disturbances on SC acquisitions, the average temperature of the room hosting the driving simulator is kept stable at 23 °C without changing the condition of humidity. Each of the simulated driving sessions has a duration of 40 min.

A further need was to relate the level of sleepiness to the physiological changes measured in the SC signal. For this purpose, we resorted to the use of the Karolinska Sleepiness Scale (KSS), which is a 9-item questionnaire that matches verbal sentences to the psychophysical state experienced by the respondent [48]. To facilitate the collection of the driver’s perceived level of drowsiness, our simulator was therefore equipped with a tablet, as shown in Figure 1. We deliberately developed a simple application in Android Studio [49] and installed it on a tablet (namely a Samsung Tab S6 Lite), which helped to easily collect the KSS value from the driver. As shown in the figure, the user interface consists of a main 3 × 3 grid and an additional large colored area in the topmost part of the screen, normally red. Each element of the grid is a clickable button corresponding to a value on the KSS scale from 1 to 9. During the test, the perceived alertness/drowsiness status is recorded periodically every 10 min, which is related to the previous time window, and to facilitate this process, the color of the top bar, which is red for most of the period, turns green to indicate to the participant to provide the perceived KSS value. As shown in Figure 1, the tablet is placed to the left of the simulator cockpit to avoid disturbing the driver during the test but at the same time to ensure that the driver can easily notice the green button and rate his level of drowsiness.

Empatica E4 is a multi-sensor wrist-worn device [18] able to collect the user’s SC signal changes during the simulated driving through the SC electrodes on the bracelet. With the E4 device, SC is exosomatically acquired in AC: a very small amount of alternating current (maximum peak-to-peak value of 100 μS) at a frequency of 8 Hz is injected through Ag/AgCl electrodes located on the bottom side of the bracelet, and the electric conductance across the skin, expressed in μS, is recorded. The sampling frequency of the SC sensor is 4 Hz.

**Figure 2:**
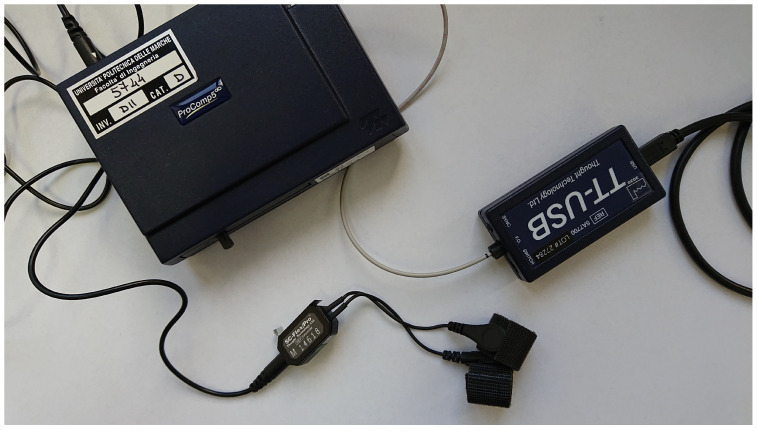
The ProComp Infiniti physiological signals DAQ. The TT-USB hardware interface and the SC sensor are shown.

For the sake of completeness, the system design also involves a second acquisition device. This is not a wearable device that can be used in substitution of the Empatica E4; rather, it has been introduced to assess the E4 acquisition capabilities and limitations in order to discover possible effects that could hinder the effective detection of drowsiness from the collected SC signals. Therefore, signals acquired from the E4 are compared to those collected by using a benchtop DAQ, namely the Procomp Infinity shown in Figure 2, which can be considered as a gold standard in the physiological measurements field. ProComp Infiniti DAQ is a high sampling frequency physiological signals acquisition system, which includes five channels, with multiple modalities: in particular, the first two channels allow the acquisition of EEG, ECG, and HR/BVP signals with a sampling frequency of 2048 Hz. The last three channels, on the other hand, allow monitoring slower signals such as respiration, temperature and SC at a frequency of 256 Hz. Channels are interchangeable, i.e., they can be used with any combination of sensors. As for the SC Flex/Pro sensor equipped with Ag/Ag/Cl electrodes, the signal input range is [0,30] μS, and the declared accuracy is ±5% and ±0.2 μS. At the hardware level, the system comprises a TT-USB interface unit, a fiber optic cable and four AA-type alkaline batteries for portable use. The ProComp system, therefore, encodes and transmits the data via the fiber optic cable to the TT-USB unit, which is in turn connected to the USB port of the PC. The companion BioGraph Infiniti software allows to easily record and export the measured values for later processing.

### 4.2. Artifacts Removal

As discussed in Section 3.2, movements of the wrist on which the sensor is worn while driving, and undesirable losses of the skin–electrodes contact, can strongly impact the quality of the SC signal and consequently decrease the accuracy of ML algorithms in detecting the driver’s drowsiness. Therefore, exploiting past experience in the scientific literature [40], the detection of changes in the SC signal typically referable to motion artifacts and their actual removal is dealt with by the Stationary Wavelet Transform (SWT) denoising with *Haar* mother Wavelet (7 levels of decomposition). In particular, according to the literature [50], the *N* Wavelet coefficients are modeled using a zero-mean Laplace distribution. Motion artifacts are removed from the samples if the corresponding coefficients fall out of the two thresholds, namely $T_{high}$ and $T_{low}$, calculated for each decomposition level, and defined as in [50] by:

(1) $$\left\{\begin{array}{c} T_{low}=(\frac{1}{N}\sum_{n=1}^{N}|d_{j}\left|\right)\;\cdot \;\log_{e}\left(\delta \right)\\ T_{high}=-T_{low} \end{array}\right.$$

where *j* is the Wavelet decomposition level, $d_{j}$ indicates the *j*-th Wavelet coefficient, *N* is the number of considered points, and δ is the proportion of motion artifacts in the original signal, thus quantifying how much motion artifacts affect the signal. The SWT must be calculated on a segment equal to $N=2^{j}$ points or on an integer multiple of *N*. In the case of the filter discussed here, we chose j=7, and the filter is applied over non-overlapped segments of $2^{7}=128$ points. As in [50,51], the value of δ is set by exploiting the information about the subject’s motion provided by the three-axial accelerometer embedded in Empatica E4, which collects wrist acceleration values simultaneously with the SC samples. Thus, δ can assume two different values depending on the magnitude of the subject’s wrist movement.

In particular, in our study, the value of δ is set based on the standard deviation (σ) of the acceleration samples collected from each single directional component ($acc_{x}$, $acc_{y}$, $acc_{z}$), as follows:

(2) $$\left\{\begin{array}{cc} \delta =0.01, & \sigma_{\left(acc_{x},acc_{y},acc_{z}\right)}<0.04\;{m/s}^{2}\\ \delta =0.10, & otherwise \end{array}\right.$$

Based on Equation (2), the limit on the value of σ has to be satisfied by all the three acceleration components. The threshold value on the acceleration, equal to 0.04 m/s^2^, is heuristically selected following a visual inspection of the time evolution of both the acceleration and SC signals. In fact, similarly to what was completed in [51], the presence of motion artifacts is identified by evaluating the σ of each acceleration component: if just one out of the three components is greater than 0.04 m/s^2^, then a motion artifact is identified and consequently removed from the signal.

After the artifact removal phase, the inverse SWT (ISWT) is applied to reconstruct the denoised signal.

### 4.3. Features Selection and Extraction

After the filtering phase, both the SC signal and its components (SCR and SCL) are divided in short-term time windows of fixed size equal to 15 s [52], each containing 60 samples acquired by the Empatica E4 device. Then, each segment is labeled with the corresponding KSS response given by the users. To investigate the drowsiness prediction capability of the SC signal variations, the KSS scores are grouped from the original 9 possible values into three classes, depending on the drowsiness level: KSS scores between 1 and 5 are grouped in class 1 (labeled as *alert*), scores equal to 6 and 7 are grouped in class 2 (labeled as *slightly drowsy*), and scores 8 and 9 are grouped in class 3 (labeled as *drowsy*). Since one recorded KSS value is referred to a period of 10 min, while the SC signal segment is 15 s long, each SC segment inside the same KSS interval has been labeled with the same KSS value. Then, from samples contained in each window, a total of 23 features (some already used in previous studies [26,53,54,55]) are initially computed in the MATLAB environment, either in time and frequency domains, to explore the temporal and spectral information content of the SC signal. For what concerns the frequency domain, before computing the selected features, Fast Fourier Transform (FFT) was applied to the original data.

Among the features extracted, however, some might have similar information content, resulting in high correlation, and hence in detrimental redundancy for discriminating classes with an ML algorithm [56]. The classification task addressed in this work exploits supervised features selection: being the class labels available, it is possible to effectively select those features that are truly discriminative in distinguishing samples from different classes. In WEKA, the so-called Correlation Attribute Evaluator performs an evaluation of each feature by computing the average Pearson’s correlation between the feature and the class. By definition, the Pearson’s correlation coefficient of two variables *X* and *Y* is calculated as:

(3) ρ=cov(X,Y)/[σ(X)·σ(Y)]

Namely, it is the covariance of the two variables divided by the product of the standard deviation (σ) of each of them. Coefficient ρ may take negative or positive values: in general, a value below −0.5 or above 0.5 is assumed to be related to a notable correlation. In this study, |ρ|> 0.90 is assumed as the condition to identify a strong correlation among the features tested and thus select only those features helpful to maximize the classifier accuracy. According to the condition set, five features, namely SC mean, SC maximum, SC median, SCL mean and SCL maximum, are discarded, thus resulting in 18 features to use, which are listed in Table 1. Finally, once computed on the acquired SC, SCL and SCR signals, each feature value is associated with the corresponding class label.

### 4.4. Machine Learning Algorithms

**Figure 3:**
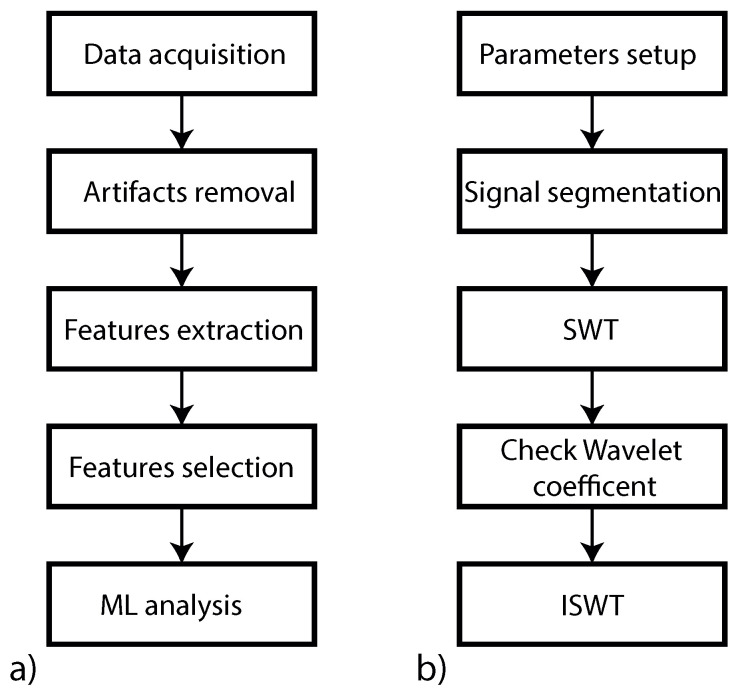
(**a**) Block scheme of the entire data processing; (**b**) Block scheme of the filter.

Once the relevant features are selected, three ML algorithms are tested and their classification performances are compared. In particular, Random Forest (RF), Bagging, and Boosting, as implemented and provided in WEKA, are considered, similarly to previous works [52,57]. The proposed ML-based drowsiness detection models are evaluated through the 10-fold cross-validation method. Then, according to [58], the classification performance is assessed based on the resulting *accuracy* (number of correctly classified instances related to driver status over the total number of instances), *precision* (number of correctly classified instances over the total number of instances labeled as belonging to the correct class), and *recall* (number of correctly classified instances over the total number of instances that actually belong to the correct class). These performance figures, expressed as percent values, are calculated as follows: (i) *accuracy* = (TP + TN)/(TP + TN + FP + FN); (ii) *precision* = TP/(TP + FP); (iii) *recall* = TP/(TP + FN). Therefore, they depend on the amount of true positive (TP), true negative (TN), false positive (FP) and false negative (FN) instances. Moreover, the confusion matrix is computed to summarize the classification performance of the resulting best ML algorithm. The aforementioned procedure for the analysis of the measured SC signals is graphically represented in Figure 3, where the right side represents a detailed block scheme of the filter designed for artifacts removal.

## 5. Filter Design Improvement

**Figure 4:**
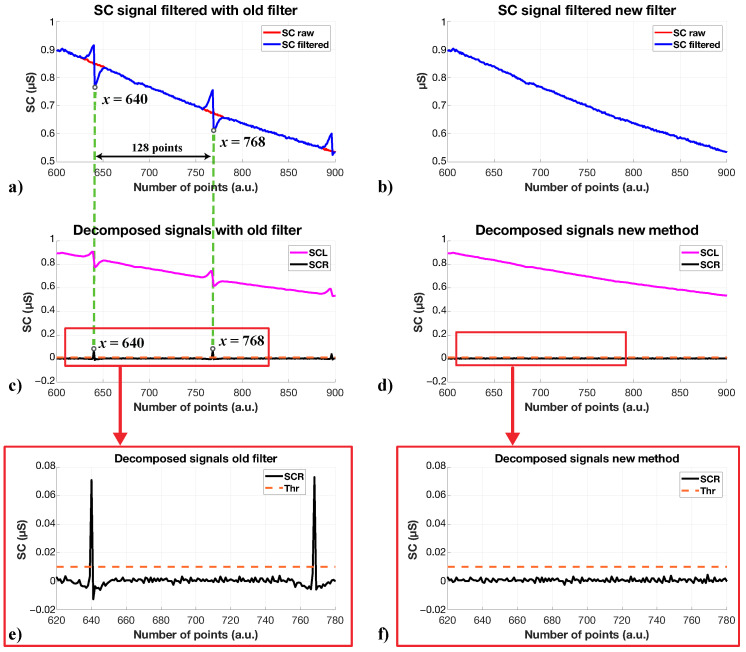
Comparison between the effect of the filter based on [51] and the modified filter. In (**a**), a portion of the acquired SC signal—the red line—that should not be affected by the filter, since it is not altered by appreciable movements, is shown. The blue line shows the signal filtered according to [51]: the visible spikes causing signal distortion are due to the ISWT; indeed, they are spaced out by 128 points, which is the length of segments the filter works on. In (**c**), the two components of the SC signal are shown (SCL in purple and SCR in black), from which several features are considered in our study. In (**e**), there is a detailed image of the SCR components where the orange dotted line is the threshold (Thr) used to consider an SCR peak relevant. The threshold is 0.01 μS, as it is usually considered. It is clear that the peaks generated by the old filter overcome the threshold and thus affect the number of peaks as a relevant feature of the SC signal. The filter distorts both components and thus the value of the features. In (**b**), the same portion of the SC signal in (**a**) is presented, but it is elaborated with the modified filter. In this case, there are no differences between the raw and filtered signals. As a result, there are no distortions of the signal components and no misinterpretations of features, as represented in (**d**) and in (**f**).

As discussed in Section 4.2, to remove motion artifacts due to the driver’s wrist movements while simulating driving, we introduced a specific filtering stage. This process relies on the SWT: in particular, it considers non-overlapped segments of the signal and works on them. We have observed that the inverse SWT (ISWT) step used at the end of the process to reconstruct the denoised signal often would create two peaks, at the beginning and the end of the considered signal segment, as shown in Figure 4. We can easily notice the effect of ISWT: peaks are equally spaced with a period equal to the length of the considered signal segment, but their magnitude differs from peak to peak. Unfortunately, these peaks modify the signal, influencing all the analytical processes, and therefore, the correct driver status assessment. For example, the number of peaks over a certain period of time is one of the most used parameters to analyze SC signals, and hence the application of the filter discussed in Section 4.2 modifies such a parameter.

**Figure 5:**
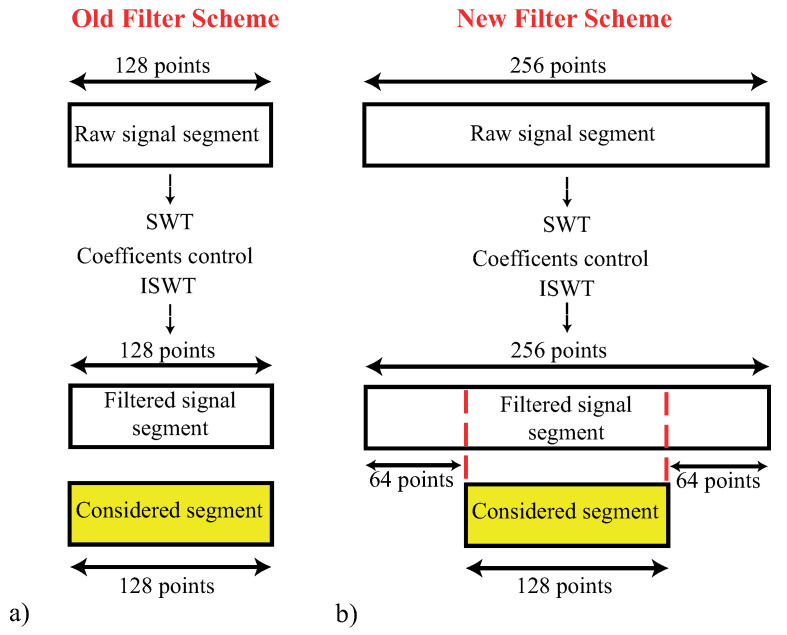
Comparison of the old and the new filter with a Wavelet decomposition level, *j*, equal to 7: (**a**) the old filter where the entire elaborated signal is considered at the end as a filtered signal; (**b**) the new filter where the filtered signal is the middle part of the elaborated one.

An improved version of the filter has been studied to overcome this issue. As shown in Figure 5, the modified filter is based on the same structure as the original one: SWT is used to remove motion artifacts, thresholds are calculated similarly, and the analysis process is applied to signal segments. However, in the new version, the segments have a length of $N=2\;\cdot \;(2^{j})$ samples, with *j* being the Wavelet decomposition level. Furthermore, in this case, the segments were overlapped with an overlapping window equal to $(2^{j})/2$. In the filtering process, during the last step, only the central part of the signal segment is considered. This way, the head and tail of each segment are not retained, and the peaks generated by the ISWT are not considered, either. As a result, the motion artifacts are removed without any additional distortion.

## 6. Experiments and Analysis of Results

For the experimental assessment carried out in this work, a set of volunteers has been constituted, which is composed of nine healthy subjects, four males and five females. As it is well-known from the literature, the age and gender of subjects can highly influence the changes in their physiological data, especially in the case of SC [59]. Therefore, a male and a female were selected in each cohort of 10 years width, from 20 to 60 years of age, to represent a variety of active drivers. Increasing the dimension of the test population would allow us to obtain a more refined representation of drivers’ behavior as well as include also those age groups that were not included in this study.

Following data acquisition and motion artifacts removal according to the improved filter design, the significant features listed in Table 1 were extracted. Then, three classification algorithms were tested. In particular, we used the WEKA tool to test the ability of Random Forest, Boosting and Bagging algorithms to detect the driver’s drowsiness level. The modified filter described in Section 5 was used with a decomposition level equal to 10 since, as described in [50], the Wavelet decomposition level is defined by:

(4) $$j=\log_{2}f_{s}+2\;,$$

where $f_{s}$ is the sampling frequency of the acquisition device and *j* is the decomposition level of the Wavelet to be used. E4 has a sampling frequency of $f_{s}=4$ Hz; thus, *j* is 4, whereas ProComp DAQ has $f_{s}=256$ Hz; thus, *j* is 10.

### 6.1. Drowsiness Classification: Comparison between Procomp and E4 SC Signals

As a first step of our experimental assessment, a detailed comparison of the signals acquired by both the E4 and the ProComp devices was necessary in order to verify the impact that different technical specifications have on the final classification outcomes. To this aim, the focus has been on the obtained signal features rather than the overall confidence about drowsiness detection. In this work, nine healthy subjects were involved (four males and five females) of different ages and gender to avoid physiological influences, as in [13]. Since Procomp Infinity is quite invasive, to maintain the ergonomics of the experiment and to avoid influences on physiological signals due to the discomfort caused by the probes of Procomp Infinity, we collected another smaller dataset to compare the classifications performance of Procomp and E4 signals. Therefore, using the driving simulator shown in Figure 1, two more volunteers were engaged: one male and one female, both healthy subjects, 28 years old. Drivers wore the E4 in the dominant hand wrist and the ProComp SC probe at the index and middle fingers of the same hand. Signals were acquired simultaneously and in the same body position in order to guarantee their coherence. Moreover, this condition allows assuming that the movements of the fingers and wrist are coherent as well. This way, the acceleration signals provided by the E4 can be used also for the analysis of ProComp data as an indicator of the amount of body motion affecting both acquisitions. Both volunteers repeated the test five times in a non-consecutive way to avoid the influences of drowsiness level due to overload.

Table 2 summarizes the figures used to evaluate the classification performance of the three algorithms on the subset collected. The overall accuracy refers to the ability to identify the three classes in which features were divided: *alert* (class 1), *slightly drowsy* (class 2) and *drowsy* (class 3). For ProComp Infiniti DAQ, the best results were obtained by Boosting classifier, but the differences with the other classifiers are actually very small. For E4, the best results were obtained by the RF classifier, according to all the performance figures evaluated, although all the results are very similar as for E4.

The results of Table 2 show higher performance for Procomp Infinity than E4; thus, despite the limitations related to the subset collected, it is possible to say that the signal acquired by the ProComp Infiniti board allows a more reliable detection of the driver’s drowsiness than E4. This is almost obvious, in our opinion, and the reasons could be several. The two most relevant ones are related to the measurement site and to the acquisition board. Fingers are the best measurement site for the SC signal [60] since they are rich in sweat glands and, in addition, they are very outward, which allows a better gathering of the SC variations. For this reason, most studies focus their attention on fingers. Unfortunately, acquiring SC signals from fingers means having electrodes on them. This could be invasive and not suitable for automotive applications.

Another important aspect to take into account is the acquisition board. Empatica E4 is a minimally invasive and comfortable wearable device that allows the collection of several physiological signals in the long term, featuring several hours of battery lifetime. This makes it a very useful device to acquire the driver’s physiological signals during driving without any discomfort, and it enables the driver’s status monitoring in a fully non-invasive way, as already demonstrated in [13]. However, these features are available at the expense of a trade-off between user comfort, power consumption, and data quality. Very often, portable devices such as smartwatches sacrifice their performance to reduce physical dimensions and improve battery lifetime. The E4, for example, has a sampling frequency of the SC signal of only 4 Hz, which is a design choice by the manufacturer that is justified by the slow-varying nature of the SC signal. On the other hand, ProComp Infiniti is a board specifically designed to acquire physiological signals with high accuracy, and it is often used to characterize prototypes. With respect to E4, it is not a fully portable device (and cannot be used as a wearable one), and it has not been designed with power consumption- or dimensions-related constraints in mind. ProComp Infiniti samples the SC signal with a frequency equal to 256 Hz, which is much higher than the 4 Hz of E4. This means that every second, the ProComp Infiniti collects 64 times the number of samples with respect to E4. This improves the quality of the signal and, consequently, the extracted features and ML results.

### 6.2. Drowsiness Classification by Oversampled E4 SC Signals

This research aims to investigate the ability of the SC signal to detect driver drowsiness using a commercially available wrist-worn wearable device. According to [13], the SC signal collected by E4 can give information about the driver’s status. At the same time, the results of the previous section demonstrated how using a more powerful device, with electrodes attached to the finger and with a higher sampling frequency, can provide (as easily expected) a better drowsiness detection accuracy.

Nevertheless, the goal of our research is to maintain a fully non-invasive data collection modality, which points to the exclusive use of a wearable device that would be compatible with real driving scenarios. Indeed, other solutions would interfere with the driver and the monitoring would become invasive. Therefore, in order to improve the accuracy drowsiness detection, we propose to increase the number of points per second collected by wearable E4 devices by oversampling.

Data processing is maintained the same as explained in Section 4.3, but we introduced an additional step to oversample the E4 signals and have the same number of samples as for the ProComp Infiniti signals. This step was made after data acquisition, since we did not want to modify the E4 device and its integrity, to maintain the FDA certification. To achieve this purpose, we made an interpolation before the motion artifacts removal phase. The interpolation was implemented in the MATLAB environment, as for the other pre-processing phases, by using the interp1 function. This function performs the 1-D data interpolation of a 1-D vector exploiting the interpolation method requested by the user: we chose the linear method that linearly interpolates the values at neighboring points. This choice was justified by an in-depth study of all the possible methods, showing that the linear interpolation produces a signal closer to the original one. Since signals are now both sampled at 256 Hz, according to the Equation (4), the Wavelet decomposition level of the filter is set equal to 10.

The results obtained with this enhanced technique are reported in Table 3 with respect to the same tested classifiers used during the previous tests: RF, Bagging and Boosting. The best result was achieved by Boosting with an *accuracy* of 89.4%, *precision* of 89.5% and *recall* of 89.5%, but there is only a 0.1 difference among percent values with RF. For ease of comparison, Table 3 reports the results obtained using the original signal at 4 Hz provided by the Empatica E4. Please notice that the numbers in the right column of the table differ from the ones reported in Table 2, and this is because Table 3 refers to the whole dataset introduced in Section 6. It is clear that the modified filtering, joint with a proper oversampling of the E4 SC measurements, provides an improvement for all classifiers, which is similar for all the figures in the case of RF and Bagging, and more evident for Boosting.

## 7. Conclusions

This study focused on the detection of driver drowsiness based on the physiological variation of the SC recorded by a wrist-worn device. Based on a previous analysis, we developed a new version of the artifact removal algorithm that made the entire data processing more robust. We compared the wearable device, E4, with a gold standard instrument for SC monitoring. We found that a signal with more samples per second allows more efficient detection of driver drowsiness. Indeed, with the ProComp Infiniti DAQ, the accuracy of drowsiness detection was 91.3% for Random Forest, 91.7% for Boosting and 90.2% for Bagging. On the other hand, the classification accuracy obtained with E4 was 83.2%, 83.2% and 82.8% for the three algorithms. These are significant differences. Since we want to keep the data acquisition mode completely non-invasive, we applied an oversampling of the E4 signals at the beginning of the processing chain. Thus, the detection accuracy results are 89.3% for Random Forest, 89.4% for Bagging, and 88.4% for Boosting. These values are higher than the results obtained without oversampling.

The improvement with respect to the first analysis is almost 5 percent points, on average. Higher accuracy means a higher ability to distinguish the driver’s status between *alert*, *slightly drowsy* and *drowsy*. Moreover, the results have demonstrated that it is feasible to detect driver drowsiness using only SC signals acquired from a single wrist-worn device in a heterogeneous population in terms of gender and age. Additionally, the classification performance is obtained with short-term time windows, which is essential for detecting short-term events such as natural drowsiness onset. When abnormal changes in skin conductance are detected, proper timely alerts can notify the driver, such as suggesting taking a short break to rest. Based on these promising results, future activities focus on extending the experimental dataset for a more robust and statistically significant evaluation of the drowsiness detection capability of SC signals alone.

## Figures and Tables

**Table 1:** Features extracted from the SC signal and its components.

Type of Signal	Domain	Features
SC signal	Time	standard deviation (μS) minimum (μS) kurtosis (μS), skewness (μS) variance ((μS)${}^{2}$), range (μS)
	Frequency	mean (μS/Hz), standard deviation (μS/Hz) minimum (μS/Hz), maximum (μS/Hz) kurtosis (μS/Hz), skewness (μS/Hz) variance ((μS/Hz)${}^{2}$), range (μS/Hz) median (μS/Hz)
SC components	Time	SCR number of peaks SCL standard deviation (μS) SCL minimum (μS)

**Table 2:** Classification results of Procomp Infinity and E4 over the subset. Below the table, there is a summary of the motivations for the differences between the two cases.

		Procomp Infinity	E4
**Random Forest**	Accuracy Precision Recall	91.3% 91.3% 91.3%	83.3% 83.2% 83.3%
**Boosting**	Accuracy Precision Recall	91.7% 91.2% 91.2%	83.2% 83.1% 83.2%
**Bagging**	Accuracy Precision Recall	90.2% 90.2% 90.2%	82.8% 82.7% 82.8%
**Features**		SC acquired on finger Higher sampling frequency Invasive Not portable	SC acquired on the wrist Lower sampling frequency Non-invasive Portable

**Table 3:** Comparison between classification results of E4 signals oversampled at 256 Hz with the ones of E4 signals at the original 4 Hz sampling frequency.

		E4 with Oversampling at 256 Hz	E4 with Original Signals at 4 Hz
**Random Forest**	Accuracy Precision Recall	89.3% 89.4% 89.3%	84.1% 84.2% 84.1%
**Boosting**	Accuracy Precision Recall	89.4% 89.5% 89.5%	82.8% 82.8% 82.8%
**Bagging**	Accuracy Precision Recall	88.4% 88.4% 88.4%	83.2% 83.3% 83.2%
